# Decreased myostatin in response to a controlled DASH diet is associated with improved body composition and cardiometabolic biomarkers in older adults: results from a controlled-feeding diet intervention study

**DOI:** 10.1186/s40795-022-00516-9

**Published:** 2022-03-15

**Authors:** Cydne A. Perry, Gary P. Van Guilder, Tammy A. Butterick

**Affiliations:** 1 Department of Applied Health Science, Indiana University School of Public Health, 1025 Seventh St., Bloomington, IN 47405 USA; 2 Exercise and Sport Science Department, Western Colorado University, Gunnison, CO 81230 USA; 3 Department of Veterans Affairs, Research Service, Minneapolis VA Health Care System, Minneapolis, MN 55417 USA; 4 Department of Neuroscinece, University of Minnesota, Minneapolis, MN 55455 USA; 5 Department of Food Science and Nutrition, University of Minnesota, St. Paul, MN 55108 USA

**Keywords:** Myostatin, Aging, Body composition, Cardiometabolic health, Diet, Obesity, Sarcopenia

## Abstract

**Background:**

Elevated concentrations of myostatin inhibit muscle growth, function and strength. Myostatin is a mediator of sarcopenia and is associated with insulin resistance. For this study we tested the response of a calorie-restricted Dietary Approaches to Stop Hypertension (DASH) diet on changes in myostatin, follistatin, and mystatin:follistatin ratio levels after 12 weeks in comparison to basline in adults aged 65 years and older. Furthermore we evaluated correlations between changes in myostatin, body composition and cardiometabolic biomarkers in this cohort of older adults.

**Methods:**

This was a controlled-feeding diet intervention study in which females (*n* = 17) and males (*n* = 11) aged 65 years and older consumed either 85 g (*n* = 15) or 170 g (*n* = 13) of fresh lean beef within a standardized DASH diet for 12-weeks. Myostatin and follistatin concentrations were measured from fasted blood samples collected at 5 timepoints throughout the 12-week feeding intervention period. Correlations were assessed between changes in myostatin and follistatin levels and measures of body composition and cardiometabolic biomarkers.

**Results:**

There were no differences (*p* > 0.05) in circulating myostatin or follistatin levels between the beef intake groups. However, with beef groups combined myostatin decreased by 17.6% (*p* = 0.006) and the myostatin-to-follistatin ratio decreased by 16.5% (*p* < 0.001) in response to the study diet. Decreased myostatin was positively correlated with reductions in waist circumference (*R*^2^ = 0.163; *p* = 0.033) and fat mass (*R*^2^ = 0.233; *p* = 0.009). There was an inverse relationship between decreased myostatin and increased strength-to-weight ratio (*R*^2^ = 0.162; *p* = 0.034). The change in myostatin-to-follistatin ratio was associated with the change in skeletal muscle mass-to-fat mass ratio (*R*^2^ = 0.176; *p* = 0.026). Decreased myostatin was positively correlated with reductions in total cholesterol (*R*^2^ = 0.193; *p* = 0.012), LDL-C (*R*^2^ = 0.163; *p* = 0.031), insulin (*R*^2^ = 0.234; *p* = 0.009), and HOMA-IR (*R*^2^ = 0.248; *P* = 0.007). There was no change (*p* > 0.05) in circulating follistatin concentrations in response to the diet intervention.

**Conclusions:**

The outcomes from this study suggest that a calorie-restricted DASH diet has the potential to reduce myostatin concentrations in older adults. Furthermore these outcomes support interrelationships between myostatin, body composition and cardiometabolic health in adults aged 65 years and older.

**Trial registration:**

ClinicalTrials.gov; Identifier: NCT04127240; Registration Date: 15/10/ 2019.

## Introduction

Myostatin is a myokine primarily produced in skeletal muscle where it suppresses cellular growth and differentiation thereby inhibiting muscle mass growth. Low levels of myostatin are also detected in other tissues such as adipose and cardiac [1, 2]. Myostatin has emerged as a potential mediator of sarcopenia and is negatively related to muscle function and strength [3–6]. Myostatin concentrations are elevated in sarcopenic obesity, negatively associated with insulin sensitivity indices and positively with measures of insulin resistance [7, 8]. Sarcopenia is primarily a disease of the older adult population and is an important determinant of muscle strength and physical performance. Additionally, older adult populations within the United States are highly susceptible to metabolic disorders such as obesity and type-2 diabetes [9]. Because older adults display co-existing factors related to sarcopenia, obesity and diabetes, they are particularly vulnerable to the negative effects of myostatin [10].

With aging body composition changes occur that include increases in body fat with concurrent decreases in muscle mass and strength. Moreover, the increase in body fat is distributed predominately within the abdominal area increasing the risk for metabolic disorders such as insulin resistance, hyperglycemia, visceral adiposity, dyslipidaemia and hypertension [11–13]. Cardiometabolic disease describes the clustering of such metabolic abnormalities and adults aged 65 years and older are at increased risk for cardiometabolic disease due to experiencing multiple related metabolic disorders thereby enhancing the onset of cardiovascular disease and type 2 diabetes [14].

What is known about the relationship between diet and myostatin is from rodent studies that involve high-fat diets, myostatin inhibitors and knock-out mouse models [15–17]. The impact of diet on changes in myostatin concentrations and parallel changes in muscle and metabolic health in humans remains unexplored. In a controlled-feeding dietary intervention study in which adults aged 65 years and older consumed a calorie-restricted Dietary Approaches to Stop Hypertension (DASH) diet for 12-weeks, we observed improved changes in body composition and biomarkers of cardiometabolic health characterized by decreases in waist circumference and fat mass, muscle strength maintenance with an increase in strength-to-weight ratio, reduced cholesterol and improved insulin sensitivity [18, 19]. Extending the scope of these findings and given the role that myostatin plays in muscle and metabolic health, we sought the following: (i) to evaluate the changes in circulating myostatin levels in response to a calorie-restricted DASH diet in older adults; (ii) to assess associations between myostatin, body composition and cardiometabolic biomarkers in this cohort of older adults; and (iii) considering the role that follistatin plays as an antagonist of myostatin, we assessed the changes in follistatin concentrations in response to the diet intervention. Considering that the relationship between diet and myostatin in humans remains relatively unknown, this study aims to contribute to this unexplored gap in knowledge.

### Participants and methods

#### Study participants

Subject characteristics, recruitment, and study diet have been previously reported [18, 19]. Briefly, older sedentary adults (> 65-years) were recruited from Brookings, South Dakota between June 2017 to August 2018. A questionnaire that included date of birth, medication use, vitamin/mineral use, and drug & alcohol use was completed by participants prior to the start of the study. Inclusion on this study was based upon: 1) age; 2) mobile ability; 3) consumption of one meal per day at the study location; 4) no comsumption of foods or beverages outside of those provided by research personnel; and 5) provide fasted blood samples at 5 timepoints throughout the intervention period. Individuals with physical and/or mobility impairments, under the age of 65 years, or could not maintain the dietary regimen/protocol were unable to participate in the study. A full characterization of body composition and cardiometabolic outcomes have been previously published [18, 19]. The study was conducted in accordance with the Declaration of Helsinki. The protocol was reviewed and approved by the Institutional Review Board for Human Study Participant Use at South Dakota State University (Approval #: IRB-1712006-EXP) and informed consent was obtained from all participants before entry into the study.

#### Study design and sample collection

As previously described, this was a parallel designed controlled-feeding diet intervention study [18, 19]. Upon entry into the study, females (*n* = 17) and males (*n* = 11) were assigned to consume either 85 g (3 oz.; *n* = 15) or 170 g (6 oz.; *n* = 13) of lean fresh beef per day within a standardized DASH-like diet [20]. Beef intake assignment for each participant was determined by random number generator (random.org) and assigned by a study investigator. The daily caloric intakes were determined using the 2015–2020 *Dietary Guidelines for Americans* for caloric intake in sedentaty older adults [21]. The composition of the study diet has been previously reported [18, 19] and was created using Nutritionist Pro software (Axxya Systems, Redmond, WA, US).

As previously described, five fasting blood samples were collected throughout the 12 week intervention period [19]. Blood was collected into EDTA-coated tubes (Pulmolab) and serum separator clot activator tubes (SST Vacutainer; Pulmolab). The SST tubes were kept at room temperature, allowed to clot, and centrifuged at 650×g for 15 min at room temperature. The EDTA-coated tubes were put on ice directly after blood collection and centrifuged within 90 min at 1055×g for 15 min at 4 °C. All samples were aliquoted into 1.8-mL cryostat vials (CryoTube; NUNC) and stored at − 80 °C.

#### Myostatin and follistatin analysis

Quantification of myostatin and follistatin were performed by the Human Nutritional Chemistry Service Laboratory at Cornell University (Ithaca, NY) [19]. Myostatin was measured using the human quantikine myostatin immunoassay solid phase enzyme-linked immunosorbent assay (ELISA; R&D systems, Minneapolis, Minnesota, USA). The human follistatin quantikine ELISA kit (R&D systems, Minneapolis, Minnesota, USA) was used to measure follistatin concentrations. Myostatin intra- and interassay CV was 3.02 and 3.32%, respectively. The intra- and interassay CV for follistatin was 2.98 and 3.43%, respectively.

#### Body composition and cardiometabolic measurements

Body composition and cardiometabolic measurements were previously detailed and reported [18, 19]. Briefly, abdominal waist circumference was measured using a Gulick tape. Bioelectrical impedance (InBody 270, InBody USA, Cerritos, California) was used to measure absolute fat mass and skeletal muscle mass. The maximum grip force of the right and left hand using a hand-held dynamometer (Smedley III analog) was used to quantify handgrip strength.

Quantification of total cholesterol, low density lipoprotein cholesterol (LDL-C) and insulin were performed by the Human Nutritional Chemistry Service Laboratory at Cornell University (Ithaca, NY) and has been previously described [19]. Briefly, the Dimension Xpand plus integrated chemistry automated analyzer (Siemens Healthineers, Malvern, Pennsylvania) was used to measure total cholesterol and LDL-C. The Immulite 2000 automated immunoassay system (Siemens Healthineers, Malvern, Pennsylvania) was used to measure insulin. Concentrations of the above biomarkers in response to the intervention have been reported [19].

The formula used to calculate the homeostatic model assessment of insulin resistance (HOMA-IR) is: fasting plasma glucose (mmol/l) times fasting serum insulin (μIU/mL) divided by 22.5 [22].

#### Statistical analysis

The statistical analysis plan for the present study is an extension of the original studies [18, 19] (ClinicalTrials.gov; Identifier: NCT04127240). An Independent Samples T test was used to assess differences in baseline characteristics, and myostatin, follistatin, and the myostatin:follistatin ratio between males and females. Differences between beef intake groups at week 12 was determined by Independent Samples T test. A linear mixed models analysis was applied to determine changes in the primary outcome variables across the intervention. A random intercept for each participant and Time (week 0, 3, 6, 9, and 12) as the fixed effect was used. The primary outcome of interest was the difference between baseline and week 12 for plasma concentrations of myostatin, follistatin, and the myostatin:follistatin ratio. When indicated by a significant Time effect, the Bonferroni adjustment for multiple comparisons was used to determine pairwise differences at specific time points. To adjust for the influence of changes in body weight across the intervention on the primary outcome variables, we repeated the linear mixed model analyses by including body weight as a covariate. A sensitivity analyses was also completed with the exclusion of the three normal weight subjects. Data for males and females are pooled but also displayed separately by sex. Relations between the change from baseline in myostatin concentrations and cardiometabolic variables, and body composition variables were determined by Pearson’s correlation coefficient. To identify the independent determinants of the change from baseline in myostatin levels, we performed stepwise multiple regression analysis. In each multiple regression model, variables with a related probability of greater than 0.10 were removed. Statistical significance was set at *p* < 0.05. Data are presented as means (SD) and analyzed with SPSS version 24 (IBM Inc., Armonk, NY, USA).

Sample size calculations for this study are based on the original study [18] which was to investigate muscle strength with varying amounts of meat. Additional studies, such as the present analysis are extensions of the original study (ClinicalTrials.gov; Identifier: NCT04127240). Thus, sample size was estimated based on an expected 22% improvement in the sit-to-stand test following 6-weeks of a high protein diet in 16 elderly adults. Using these estimates, sample sizes were calculated based on 80% power at an alpha level of 0.05 to detect an absolute mean improvement of 4 ± 3 repetitions in the 30-s sit-to-stand test. Resulting sample sizes were 14 participants per group, respectively.

## Results

### Baseline (wk 0) characteristics of study participants

**Fig. 1:**
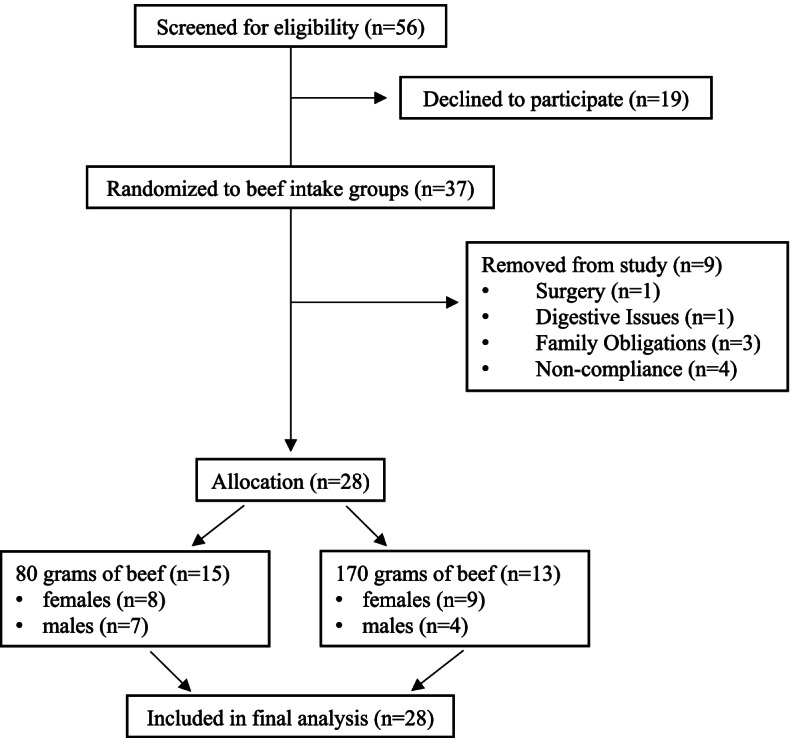
Schematic of Particiapnt Flow

Twenty-eight participants aged 65–84 years completed the 12-week diet intervention and were included in the final analysis (Fig. 1). As previously described [18, 19], all participants presented with the following at baseline: waist circumference: 101 ± 16.4 cm; absolute fat mass: 34.5 ± 12.7 kg; grip strength: 0.70 ± 0.21 per kg body mass; skeletal muscle mass: 31.4 ± 7.2 kg; total cholesterol: 180.9 ± 38.1 mg/dL; LDL-C: 104.5 ± 28.9 mg/dL; insulin: 14.1 ± 8.2 μIU/mL; HOMA-IR: 4.0 ± 3.3.

Baseline characteristics of study participants separated by meat intake groups are presented in Table 1. There were no statistically significant differences (*p* > 0.05) detected between the 85 g and 170 g meat intake groups at baseline for age (*p* = 0.834), myostatin (*p* = 0.326), follistatin (*p* = 0.569) or myostatin:follistatin ratio (*p* = 0.402).

**Table 1:** Baseline characteristics of study participants separated by meat intake group

Variables	85 g meat intake group (***n*** = 15)	170 g meat intake group (***n*** = 13)
Age (years)	70.6 (5.9)	71.1 (6.0)
Female	8	9
Male	7	4
Myostatin (ng/mL)	3.15 (1.41)	3.78 (1.94)
Follistatin (ng/mL)	1.66 (0.44)	1.75 (0.46)
Myostatin:Follistatin	1.94 (0.83)	2.32 (1.49)

Data are presented as means and standard deviations with the exception of the number of females and males

Baseline characteristics of study participants separated by sex are presented in Table 2. Statistically significant differences (*p* > 0.05) between males (*n* = 11) and females (*n* = 17) were not detected for myostatin (*p* = 0.2), follistatin (*p* = 0.12) or myostatin:follistatin ratio (*p* = 0.536). Baseline body composition characteristics and cardiometabolic measurements separated by meat intake groups and sex has been previously published [18, 19]. Briefly, there were no statistically significant differences (*p* > 0.05) at baseline for body composition measures or cardiometabolic markers separated by meat intake groups. At baseline males had greater (*p* < 0.05) body fat, waist circumference and grip strength compared to females and females had higher total cholesterol (*p* = 0.02) compared to males.

**Table 2:** Baseline characteristics of study participants separated by sex

Variables	Total (***n*** = 28)	Male (***n*** = 11)	Female (***n*** = 17)	***p*** value
Myostatin (ng/mL)	3.4 (1.7)	4.0 (1.6)	3.1 (1.7)	0.2
Follistatin (ng/mL)	1.7 (0.44)	1.9 (0.54)	1.6 (0.34)	0.12
Myostatin:Follistatin Ratio	2.12 (1.17)	2.29 (1.16)	2.00 (1.21)	0.536

Data are presented as means and standard deviations. Independent samples T test was performed to determine sex differences

### Myostatin and follistatin changes in response to the study diet

Myostatin and follistatin concentrations in response to meat intake at week 12 are presented in Table 3. By week 12 of the intervention there were no statistically significant differences (*p* > 0.05) between the 85 g and 170 g meat intake groups on myostatin (*p* = 0.206), follistatin (*p* = 0.819) or myostatin:follistatin ratio (*p* = 0.369).

**Table 3:** Myostatin and follistatin concentrations of older adults at week 12

Variables	85 g meat intake group (***n*** = 15)	Percent change from baseline	170 g meat intake group (***n*** = 13)	Percent change from baseline	***p***-value
Myostatin (ng/mL)	2.59 (0.96)	−15.0 (10.6)	3.14 (1.31)	−12.4 (20.9)	0.206
Follistatin (ng/mL)	1.68 (0.48)	5.1 (28.6)	1.73 (0.65	−3.3 (15.8)	0.819
Myostatin:Follistatin	1.64 (0.76)	−14.1 (18.9)	1.94 (1.00)	−8.1 (26.6)	0.369

Data are presented as means and standard deviations. Independent samples T test on absolute data was performed to determine group differences in myostatin, follistatin, and the myostatin-to-follistatin ratio by meat intake group

Myostatin and follistatin responses to the intervention diet with both meat intake groups combined are shown in Table 4. Throughout the 12-week intervention period, responses to the study diet during the intervention period were detected for myostatin (*p* < 0.001) and the myostatin:follistatin ratio (*p* < 0.001) such that a decrease was observed. In response to the study diet, myostatin decreased by 17.6% (*p* = 0.006) from baseline (3.4 ng/mL) to study-end (2.8 ng/mL). Myostatin:follistatin ratio decreased by 16.5% (*p* = 0.001) from baseline (2.12 ng/mL) to study-end (1.77 ng/mL). Follistatin concentrations did not change (*p* = 0.130) from baseline (1.7 ng/mL) to study-end (1.7 ng/mL) in response to the intervention diet. Sensitivity analyses also revealed that the significant decrease in myostatin concentrations remained after excluding the normal weight subjects.

**Table 4:** Myostatin and follistatin concentrations in older adults consuming the DASH diet for 12 weeks

	Weeks of Intervention					
Variable	0	3	6	9	12	***p***-value
Myostatin (ng/mL)	3.4 (1.7)*	3.0 (1.5)*	2.9 (1.2)*	2.9 (1.3)*	2.8 (1.1)*	0.006
Follistatin (ng/mL)	1.7 (0.44)	1.6 (0.47)	1.6 (0.48)	1.6 (0.41)	1.7 (0.55)	0.130
Myostatin:Follistatin Ratio	2.12 (1.17)*	2.01 (1.17)	1.96 (0.92)	1.96 (1.11)	1.77 (0.87)*	< 0.001

Data are presented as means and standard deviations. Linear mixed models with fixed effect of Time wks 0, 3, 6, 9, 12 and random subject and intercept for each participant was used to determine changes in the primary outcome variables across the intervention. *p < 0.05 versus baseline

### Correlations between myostatin and measures of body composition

**Fig. 2:**
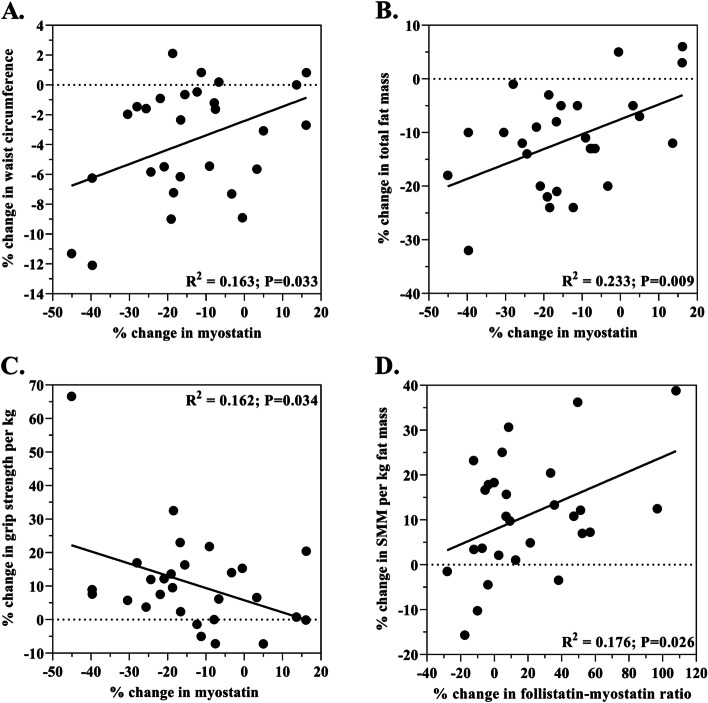
Correlations Between The Percent Decrease In Myostatin and Measures of Body Composition Across the Dietary Intervention

The 17.6% decrease in myostatin was correlated with anthropometric measures of waist circumference, absolute fat mass, grip strength and muscle mass (Fig. 1). The decrease in myostatin was correlated with decreases in waist circumference (*R*^2^ = 0.163; *p* = 0.033) and absolute fat mass (*R*^2^ = 0.233; *p* = 0.009) (Fig. 2A and B). The decrease in myostatin was correlated with the increase in the grip strength-to-weight ratio (*R*^2^ = 0.162; *p* = 0.034) (Fig. 2C). The 16.5% change in the myostatin:follistatin ratio was correlated with the change in skeletal muscle mass per kilogram of fat mass (*R*^2^ = 0.176; *p* = 0.026) (Fig. 2D).

### Associations between myostatin and cardiometabolic biomarkers

**Fig. 3:**
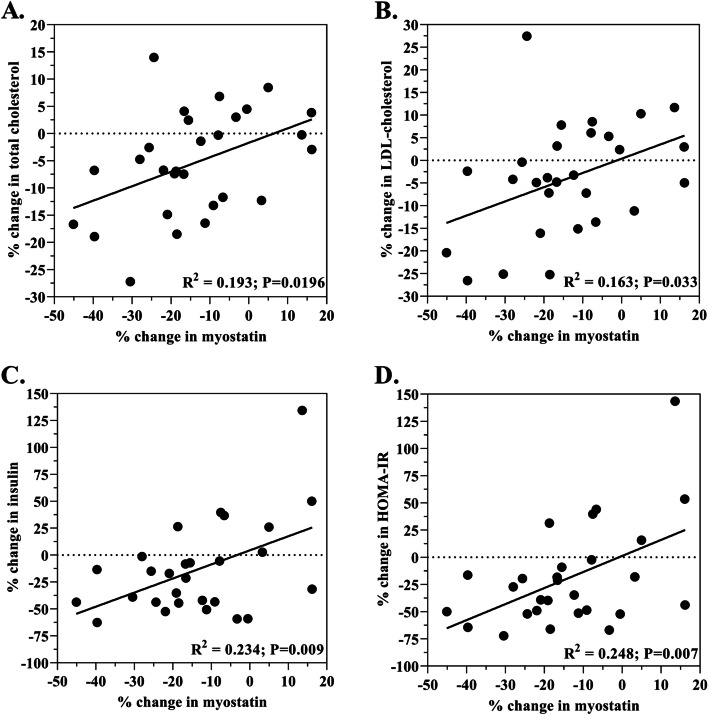
Correlations Between The Percent Decrease in Myostatin and Cardiometabolic Biomarkers Across The Dietary Intervention

The 17.6% decrease in myostatin was correlated with decreases in total cholesterol (*R*^2^ = 0.193; *p* = 0.0196); LDL-C (*R*^2^ = 0.163; *p* = 0.033); insulin (*R*^2^ = 0.234; *p* = 0.009); and HOMA-IR (*R*^2^ = 0.248; *p* = 0.007) (Fig. 3).

### Independent predictors of the change in Myostatin

We included age, and the percent changes from baseline in total cholesterol, LDL-C, insulin, HOMA, body weight, percent body fat, BMI, follistatin, and grip strength in the multiple regression model to predict the change in myostatin. The prediction model was statistically significant (*F* = 8.553; *p* = 0.002) and accounted for 38% of the variance of the decrease in myostatin (Adjusted *R*^2^ = 0.377). Percent reduction in fat mass (β = 0.501; *p* = 0.009) and the percent increase in grip strength-to-weight ratio (β = − 0.419; *p* = 0.014) independently predicted the decrease in myostatin.

## Discussion

This highly controlled feeding study sought to evaluate the impact of the DASH diet on changes in myostatin and follistatin concentrations and assess whether these changes were associated with measures of body composition and markers of cardiometabolic health in a cohort of sedentary older adults. In response to the 12-week diet intervention, we observed a significant decrease in myostatin concentrations. Furthermore, relationships between myostatin, body composition changes and biomarkers of cardiovascular health and insulin sensitivity emerged.

### Myostatin decreased in older adults consuming the DASH diet and was correlated with changes in body composition

In the present study under controlled-feeding conditions, circulating myostatin levels significantly decreased in all participants by 17.6% (*p* = 0.006). There was no difference in myostatin when separated by meat intake groups (85 g vs. 170 g). The 17.6% reduction in myostatin was correlated with a 4.7% reduction in waist circumference [18] (*R*^2^ = 0.163; *p* = 0.033; Fig. 2A) and a 12.2% decrease in absolute fat mass [18] (*R*^2^ = 0.233; *p* = 0.009; Fig. 2B). Furthermore, the percent reduction in fat mass independently predicted the decrease in myostatin (β = 0.501; *p* = 0.009). The relationship between myostatin and waist circumference has been previously reported. In a cross-sectional case-control study in young women with and without polycystic ovary syndrome (PCOS), elevated myostatin levels were positively correlated with increased waist circumference in women with PCOS which may be suggestive of an increased risk for abdominal obesity [7]. Interestingly, adults with polymorphisims in the Myostatin (*MSTN)* gene display higher waist circumference and total fat mass, implying that variations in the *MSTN* gene may be an influential factor related to abdominal adiposity and obesity [23]. The relationship between elevated myostatin and obesity has been demonstrated in both rodent and human studies. For example, myostatin mRNA is increased in both skeletal muscle and adipose tissues in wild type mice fed a high fat diet and genetically obese mice [24]. Increased circulating and muscle myostatin concentrations have been reported in adults with extreme obesity (i.e. BMI of 49 kg/m^2^) and BMI is strongly correlated with muscle myostatin concentrations [25]. Conversely, reports from rodent studies show reductions in body fat levels with inhibition of myostatin either pharmacologically or through gene-knockout models [26–31]. Human trials demonstrate decreased myostatin mRNA with weight loss due to either biliopancreatic diversion or gastric bypass surgery [31, 32]. Indeed, the outcomes of the present study in a cohort of obese older adults show associations between decreased myostatin and decreasesd waist circumference and fat mass in response to the DASH diet intervention and that decreased fat mass was an independent predictor for the decrease in myostatin. Although BMI decreased in response to the study diet [18], BMI was not correlated with myostatin in the present study. Taken together, these results suggest that in addition to pharmacological and surgical methods, the DASH diet maybe a potential strategy to reduce myostatin levels with concurrent parallel changes in adiposity that may in-turn benefit individulas with obesity.

The 17.6% reduction in circulating myostatin with the DASH diet intervention was significantly related to beneficial changes in skeletal muscle mass and strength in this cohort of older adults. We observed that the decrease in myostatin was associated with improvements in grip strength-to-weight ratio (i.e., as body mass decreased over time, grip strength increased across the intervention time period; *R*^2^ = 0.162; *p* = 0.034; Fig. 2C) and skeletal muscle mass-to-fat mass ratio (i.e., as skeletal muscle mass increased over time, fat mass decreased across the intervention time period; *R*^2^ = 0.176; *p* = 0.026; Fig. 2D). Notbly, we previously reported a correlation between improved grip strength-to-weight ratio and reduced waist circumference [19], both of which are associated with reduced myostatin reported in the present study. It is evident from a number of studies that increased myostatin levels are associated with reduced skeletal muscle mass and strength in humans [5, 33–35]. Furthmore, recent estimates indicate a 7.6 increased odds of low grip strength in older adults with elevated myostatin levels [5]. Findings from this study extend these prior studies by showing that the improvement in grip strength-to-weight ratio was an independent predictor of the decrease in myostatin (β = − 0.419; *p* = 0.014). Since grip strength alone serves as the dominant predictor for poor health outcomes in older adults, the results of the present study would be of vital importance to older individulas as the outcomes suggest that decreased myostatin is associated with improved relative body strength. This is important because decreased activities of daily living and reduced walking mobility in older adults is due in part to poor body strength (i.e. decreased strength-to-weight ratio and muscle mass). Poor body strength increases the risk of falls that in-turn lead to mobile disability in older adults. By improving the grip strength to weight ratio, older individuals may able to perform activities of daily living, including walking, bathing and stair climbing at a lower percentage of their strength capacity, thus, reducing the physical effort required to perform everyday activities. The association between decreased myostatin and improved skeletal muscle mass-to-fat mass ratio observed in the present study may be explained, in part, by an indirect effect of increased muscle mass and/or loss of myostatin signaling in muscle rather than an effect of the loss myostatin signaling in adipose tissue [28].

From a cardiometabolic health perspective, these outcomes are critical given that skeletal muscle is the largest consumer of glucose (> 80% of insulin-mediated glucose metabolism occurs in skeletal muscle [36]) and plays a central role with insulin sensitivity. Thus, the reductions in myostatin with the DASH diet coupled with improvements in skeletal muscle mass and strength, may underlie the beneficial changes in cardiometabolic health and insulin sensitivy observed in the present study.

### Decreased myostatin concentrations are associated with decreased concentrations of total cholesterol, LDL-C, insulin and HOMA-IR

Outcomes of the present study show that in response to the study diet the 17.6% reduction in myostatin was associated with reductions in total cholesterol (*R*^2^ = 0.193; *p* = 0.0196; Fig. 3A) and LDL-C (*R*^2^ = 0.163; *p* = 0.033; Fig. 3B). It is well-known that total cholesterol and LDL-C serve as biomarkers in the lipid profile for cardiovascular disease [37]. However the role that myostatin plays with these biomarkers remains unknown. A rodent study using a myostatin/LDL-C double-knockout mouse model reported reduced LDL-C concentrations along with reduced free fatty acids and triglycerides in response to a high-fat/high cholesterol diet compared to the LDL-C knockout control [38]. In a human study, however, a negative relationship was reported in-which decreased LDL-C was associated with increased myostatin in women with PCOS [39]. Results from the present study show that in response to the DASH diet intervention decreased myostatin was correlated with decreased LDL-C in a cohort of obese older adults. With conflicting results and very few reports, more studies are required to understand the relationship between myostatin and cholesterol.

In the present study, the 17.6% decrease in myostatin was associated with a 13.4% decrease in insulin [19] (*R*^2^ = 0.234; *p* = 0.009; Fig. 3C) and 25% reduction in HOMA-IR [18] (*R*^2^ = 0.248; *p* = 0.007; Fig. 3D). Moreover, we previously reported a 8.4% decrease in fasted blood glucose concentrations and correlations between decreased waist circumference and decreased insulin and HOMA-IR [19]. While it is well-established that a primary action of myostatin is to inhibit muscle growth, new evidence suggests it can also play a key role contributing to metabolic dysfunction and insulin resistance [25, 28, 40, 41]. For example, myostatin expression in skeletal muscle was reported to be negatively associated with insulin sensitivity in adults aged 54–77 years described as either overwight or obese [41]. Additionally, increased myostatin in muscle has also been reported in metabolic conditions associated with insulin resistance such as type 2 diabetes [31] and dysglycemia [40]. These findings are consistent with those of Hittel et al. [25] who showed that greater myostatin protein content in skeletal muscle of obese adults was associated with worse HOMA-IR. Other in vitro studies have shown that myostatin inhibits glucose uptake in a dose-dependent manner in placental cell lines [42]. Conversely, disruption of the myostatin gene in a high-fat diet mouse model prevents diet-induced obesity and insulin resistance [27]. Intriguing findings by Guo et al. [28] showed that myostatin knockout mice displayed greater insulin sensitivity compared to wild type mice. Interestingly, this outcome remained after controlling for increased muscle mass, implying that myostatin may have unfavorable effects on insulin sensitivity that are independent of reduced muscle mass. This could potentially exacerbate the metabolic effects of sarcopenic obesity given the decreased glucose uptake resulting from decreased muscle mass heightened by the inhibitory action of myostatin on insulin sensitivity. Collectively, these studies and others [26, 43] point to a growing body of evidence that increased myostatin is involved in contributing to metabolic dysfunction in the skeletal muscle of obese individuals, particularly when obesity is associated with metabolic abnormalities, suggesting that it may contribute to insulin resistance [25].

Interventions that have reported declines in skeletal muscle myostatin with improvements in insulin sensitivity and decreased body fat include gastric bypass surgery and exercise training [27, 38]. In muscle isolated from morbidly obese women before and one year after gastric bypass surgery, myostatin expression significantly decreased by 2.4-fold [32]. This reduction in skeletal muscle myostatin was significantly related to improved insulin sensitivity. In a 12 week intervention that included aerobic exercise plus dietary restriction in middle-aged and older adults resulted in a decrease in skeletal muscle myostatin and body fat and increased insulin sensitivity [41]. Unfortunately, outcomes regarding muscle mass or strength were not reported. Similar to these findings we report that in response to a 12 week calorie-restricted DASH diet intervention (with no exercise intervention) in adults aged 65 years and older circulating myostatin concentrations decreased and were associated decreased fat mass as well as improved insulin sensitivity as measured by decreased circulating insulin and HOMA-IR. Extending the knowledge of the prior study, we also report improvements muscle mass and strength in relation to reduced myostatin. Notably, we report that reduced fat mass and improved strength independently predict for the decrease in myostatin. Although the participants in the present study lost 6.3% of total body mass in response to the diet intervention [18], when adjusting for weight loss in the present study, the significant decreases in myostatin remained. Taken together, these outcomes are suggestive of a primary modulatory influence of the DASH diet on myostatin and imply that the DASH diet may be a positive effective intervention in the older adult population.

### Limitations

Limitations of this study include: (i) a control or comparison group was not tested; (ii) all participants self-identified as white American which represents the dominant ethnic group in South Dakota; (iii) all participants lived in their own homes and were mobile; (iv) no one resided in assisted living facilities and support was not required for daily living activities. These limitations should be considered when generalizing the outcomes of the present study to various populations of older adults including those with diverse ethnic and demographic backgrounds as well as different living conditions.

## Conclusion

Results from this controlled-feeding diet intervention study suggest that a calorie-restriced DASH diet has the ability to effectively reduce myostatin concentrations in adults aged 65 years and older. Moreover, the outcomes of the present study show that decreased myostatin levels are paired with improvements in muscle and cardiometabolic health and that muscle strength and fat mass may serve as independent predictors for myostatin concentrations. Results from this study also show that follistatin did not respond to the intervention diet. Given that the effect of diet on myostatin in humans remains largely unexplored, additional studies are required to determine the long-term clinical effects of diet on myostatin, muscle health and insulin sensitivity. Although correlations emerged between myostatin and cardiometabolic biomarkers, further studies are needed to elucidate the clinical relevance of these relationships for the preservation of age-related muscle loss and metabolic disease.

## Data Availability

The datasets used and analysed for the current study are available from the corresponding author upon reasonable request.

## Abbreviations

DASH: Dietary Approaches to Stop Hypertension
ELISA: Enzyme-linked immunosorbent assay
HOMA-IR: Homeostatic model assessment of insulin resistance
LDL-C: Low density lipoprotein-cholesterol
oz.: ounces
PCOS: Polycystic ovary syndrome
SMM: Skeletal muscle mass
